# circ-Pank1 promotes dopaminergic neuron neurodegeneration through modulating miR-7a-5p/α-syn pathway in Parkinson’s disease

**DOI:** 10.1038/s41419-022-04934-2

**Published:** 2022-05-19

**Authors:** Qing Liu, Qiyao Li, Runjiao Zhang, Hongfang Wang, Yibo Li, Ziyu Liu, Wenmeng Xie, Dandan Geng, Lei Wang

**Affiliations:** 1 Department of Human Anatomy, Institute of Medicine and Health, Hebei Medical University, Shijiazhuang, Hebei 050017 China; 2 Department of Emergency Surgery, The Second Hospital of Hebei Medical University, Shijiazhuang, 050000 China; 3 The Key Laboratory of Neural and Vascular Biology, Ministry of Education, Hebei Medical University, Shijiazhuang, Hebei 050017 China

**Keywords:** Parkinson's disease, Non-coding RNAs

## Abstract

Circular RNA (circRNA) is a type of non-coding RNA that is widely expressed in mammals. It is highly conserved and abundantly expressed in the brain. Here, we report the regulatory role of circRNA derived from the pantothenate kinase 1 (Pank1) gene (circ-Pank1) in Parkinson’s disease (PD). Circ-Pank1 is highly expressed in the substantia nigra (SN) of PD model mice treated with rotenone and in the MN9D cell model of dopaminergic neurons. The circ-Pank1 knockdown ameliorated dopaminergic neuron damage and locomotor dysfunction after the treatment with rotenone. We found that circ-Pank1 could adsorb miR-7a-5p and upregulate the expression of α-synuclein (α-syn), which is a molecular hallmark closely related to PD. The inhibition of miR-7a-5p reversed the circ-Pank1 knockdown-induced amelioration of dopaminergic neuron injury. In conclusion, circ-Pank1 is overexpressed in PD and enhances the locomotor dysfunction via the miR-7a-5p/α-syn signaling axis. We revealed the functional role of circRNAs in the progression of PD and provided a potential target for noncoding RNAs in delaying the progression of PD.

## Introduction

Parkinson’s disease (PD) is a progressive neurodegenerative disease characterized by the selective loss of dopaminergic neurons of the substantia nigra (SN) [1]. Dopaminergic neuron loss results in reduced striatal dopamine and subsequently leads to the most prominent motor symptoms of the patients, including bradykinesia, rigidity, resting tremor, and gait disturbance with postural instability [2]. The major molecular hallmark of PD is an intracellular accumulation of protein deposits (named Lewy bodies), which are primarily composed of precipitates of the α-synuclein (α-syn) protein [3, 4], so understanding how to block its ability to cause neurodegeneration may be beneficial for PD patients.

Circular RNAs (circRNAs) are a distinctive class of noncoding RNAs characterized by the presence of alternative splicing, most commonly in which the splice donor site of one exon is ligated to the splice acceptor site of an upstream exon [5–7]. They are evolutionarily conserved molecules and specifically enriched in the nervous system [8, 9]. Emerging evidence indicates that circRNAs are dynamically modulated and have important regulatory functions in neurodegenerative diseases and aging-related diseases [10, 11]. Recent studies have focused on evaluating the role and mechanism of circRNAs in human diseases as a method to develop new treatment strategies. CircRNAs have been identified as important molecules in gene expression regulation at the post-transcriptional level. There is evidence that some circRNAs may regulate miRNA functions, and the roles in transcriptional control have been suggested [12, 13]. CircRNAs were reported to function in gene regulation by competing with linear splicing [14].

Nuclear factor erythroid 2-related factor (Nrf2) function in the central nervous system was initially shown to bind to the antioxidant responsive element (ARE) and activate a program of gene expression that mitigated reactive oxygen species and the damage that ensued [15]. Nrf2 mitigates neurodegeneration in genetic and toxin models of PD [16], and loss of Nrf2 exacerbates degeneration [15], which implies that it is a potential therapeutic target of PD [17]. With the development of deep sequencing technology, more noncoding RNAs have been identified and shown to be related to brain diseases [18, 19]. Our previous study analyzed the expression of circRNAs in the SN of Nrf2^-/-^ mice compared with wild-type mice using a full transcriptome microarray; according to the large difference and small *P* value, four differentially expressed circRNAs were screened [20].

In this study, we tested the expression of the four differentially expressed circRNAs in PD model mice treated with rotenone by qRT–PCR and found that mmu_circRNA_32463 increased most significantly and derived from the pantothenate kinase 1 (Pank1) gene, so we named it circ-Pank1. We further found that circ-Pank1 knockdown ameliorated the rotenone-induced dopaminergic neuron injury and detected the underlying mechanisms. Circ-Pank1 upregulated the α-syn expression by competitively adsorbing miR-7a-5p. This study describes a novel circRNA-α-syn regulatory mechanism and provides a potential therapeutic target for PD.

## Result

### Circ-Pank1 is increased in the PD model treated with rotenone

**Fig. 1:**
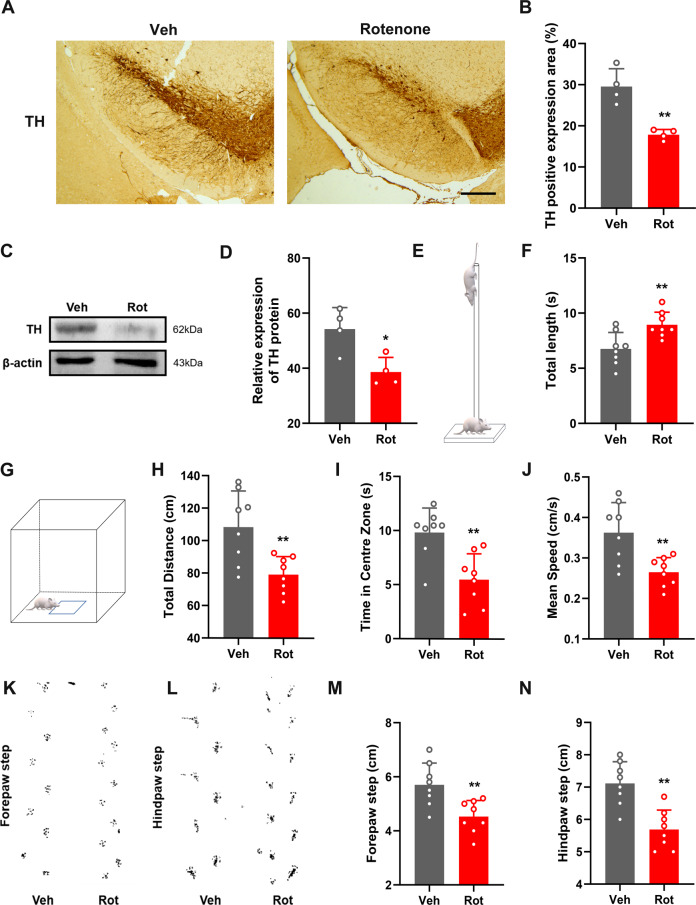
Mice treated with rotenone showed PD-like pathological changes and motor dysfunction. **A**, **B** Number of TH-positive cells in the brain tissue of PD model mice treated with rotenone; scale bars, 500 μm (*n* = 4). **C**, **D** Western blot analysis of the TH protein levels in the SN of PD model mice treated with rotenone (*n* = 4). **E**–**N** Pole-climbing test **E**, **F**, field test (**G**–**J**) and step measurement **K**–**N** were performed on PD model mice treated with rotenone to evaluate the exercise ability (*n* = 8). Data are presented as mean ± SD. *P < 0.05, **P < 0.01.

To confirm the dopaminergic degeneration caused by rotenone treatment, we first assessed the PD-like pathology and motor dysfunction in ICR mice after intragastric administration of rotenone (30 mg/kg) for 4 consecutive weeks. A significant reduction in tyrosine hydroxylase (TH)-positive neurons was observed in the SN (Fig. 1A–D). Consistent with neuropathological and biochemical findings, motor dysfunction was observed after the rotenone treatment. In the pole test, the mice in the rotenone (Rot) group showed unsteady gait and slightly trembled during the pole climbing process, and the time spent crawling down the pole was significantly longer than that in the vehicle control (Veh) group (Fig. 1E, F). In the open field test, the Rot group travelled a significantly shorter total distance than the Veh group (Fig. 1G, H). The Rot group had significantly less residence time in the central area and average speed than the Veh group (Fig. 1I, J). The step measurement results showed that the step distances of the forepaw and hindpaw in the Rot group were significantly smaller than those in the Veh group (Fig. 1K–N). Overall, the mice showed a significant reduction in overall motor activity, reduced movement time and step distance in the open field test, and impaired balance and coordination with an apparent increase in climbing downtime in the pole test. The mice treated with rotenone successfully showed a PD-like pathology and motor phenotype and an impaired locomotor phenotype.

**Fig. 2:**
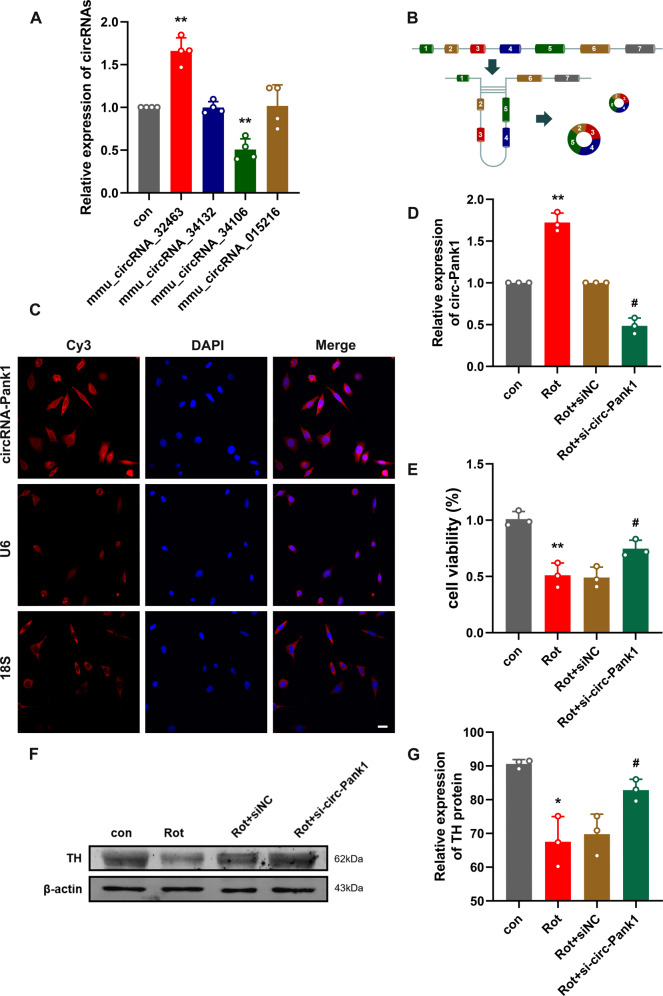
The expression of circ-Pank1 significantly increased in PD model mice treated with rotenone and downregulation of circ-Pank1 in the MN9D cell model alleviates the damage to dopaminergic neurons. **A** qRT–PCR was used to detect the expression of mmu_circ_32463, mmu_circ_34132, mmu_circ_34106, and mmu_circ_015216 in the SN of PD model mice treated with rotenone (*n* = 4). **B** Schematic diagram of ring formation of circ-Pank1. **C** Sublocalization of circ-Pank1 in MN9D cell. Taking U6 in the nucleus and 18S in the cytoplasm as the controls, most circ-Pank1 in MN9D cells was located in the cytoplasm; scale bars, 50 μm. **D**–**G** After si-circ-Pank1 and the corresponding control were transferred into the MN9D cell model treated with rotenone (*n* = 3). **D** qRT–PCR was used to detect the expression of circ-Pank1. **E** CCK-8 was used to detect the cell viability. **F**, **G** Western blot analysis of the TH protein levels. Data are presented as mean ± SD. *P < 0.05, **P < 0.01.

Our previous work analyzed the circRNA profiles in the SN of wild-type and Nrf2^-/-^ mice using a whole transcriptome microarray [20]. We selected four significantly differentially expressed circRNAs in the SN of Nrf2^-/-^ mice, tested the expression of four differentially expressed circRNAs in the PD model treated with rotenone by qRT–PCR, and found that the increase in mmu_circ_32463 was most significant (Fig. 2A). Thus, in this study, we focused on the expression and role of mmu_circ_32463 in the rotenone-induced PD model. According to UCSC data (http://genome.ucsc.edu/), mmu_circ_32463 is formed by reverse splicing of exons 2-5 of the Pank1 gene, termed circ-Pank1 (Fig. 2B). To confirm the cellular localization of circ-Pank1, fluorescence in situ hybridization (FISH) showed that circ-Pank1 was mainly located in the cytoplasm (Fig. 2C).

### Silencing circ-Pank1 inhibits the dopaminergic neuron damage caused by the rotenone treatment in vitro

To further test the functions of circ-Pank1 in the PD-like pathology and locomotor phenotype, siRNAs that target the back-splice junction sites of circ-Pank1 were designed and transfected into MN9D cells treated with rotenone. The Circ-Pank1 expression was greatly knocked down by si-circ-Pank1. The efficiency of circ-Pank1 deletion was confirmed by qRT–PCR assays. Based on the si-circ-Pank1 transfection or rotenone treatment, MN9D cells were divided into 4 groups: control (con) group, rotenone treatment (Rot) group, rotenone + negative control (Rot + siNC) group, and rotenone + circ-Pank1 siRNA (Rot + si-circ-Pank1) group. The expression of circ-Pank1 was increased in the Rot group of MN9D cells and reversed with si-circ-Pank1 transfection in the Rot+si-circ-Pank1 group (Fig. 2D). Meanwhile, the number of apoptotic cells induced by the rotenone treatment was reduced after the si-circ-Pank1 transfection (Fig. 2E). In addition, the TH expression was significantly inhibited by the rotenone treatment, but this inhibitory effect was reversed after the si-circ-Pank1 transfection (Fig. 2F, G). These findings reveal that interference with circ-Pank1 ameliorates the neuronal injury induced by the rotenone treatment.

### Circ-Pank1 promotes dopaminergic neuron injury by increasing the expression of α-syn

**Fig. 3:**
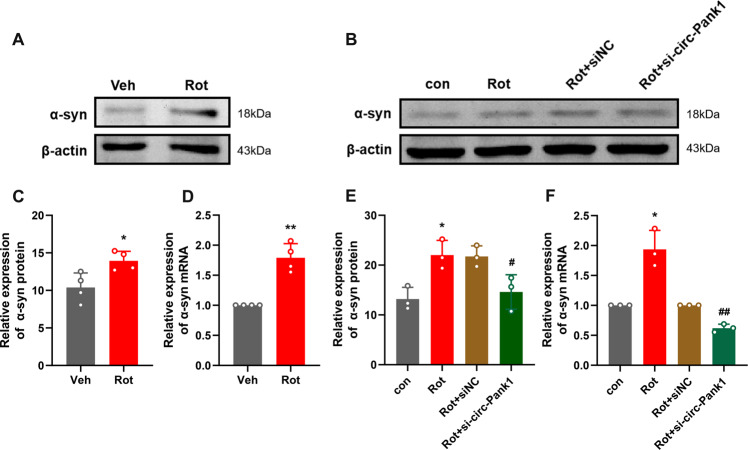
The downregulation of circ-Pank1 in the MN9D cell model alleviates the damage to dopaminergic neurons by reducing the expression of a-syn. **A**, **C** Western blot analysis of the protein levels of α-syn in the SN of PD model mice treated with rotenone (*n* = 4). **D** qRT–PCR to detect the expression of α-syn mRNA in the SN of PD model mice treated with rotenone (*n* = 4). **B**–**F** After si-circ-Pank1 and the corresponding control were transferred to the MN9D cell model treated with rotenone (*n* = 3), Western blot analysis of the protein level of α-syn (**B**, **E**) and qRT–PCR detection of the mRNA expression of α-syn **F**. Data are presented as mean ± SD. *P < 0.05, **P < 0.01.

To detect the detailed mechanism of circ-Pank1 in dopaminergic neuron loss and impaired locomotor behaviors caused by the rotenone treatment, we focused on the α-syn protein, which is involved in PD dopaminergic neuron injury. The aggregation of the α-syn protein is the pathological hallmark of PD. In the PD model mice treated with rotenone, we found that the expression of α-syn significantly increased compared with the control group (Fig. 3A, C, D). To further confirm the hypothesis that circ-Pank1 upregulates the α-syn expression, we analyzed the mRNA and protein expression levels of α-syn in MN9D cells treated with rotenone by Western blot and qRT–PCR experiments. The circ-Pank1 knockdown decreased the expression levels of α-syn protein and α-syn mRNA (Fig. 3B, E, F).

### Circ-Pank1 functions as an efficient miR-7a-5p sponge

**Fig. 4:**
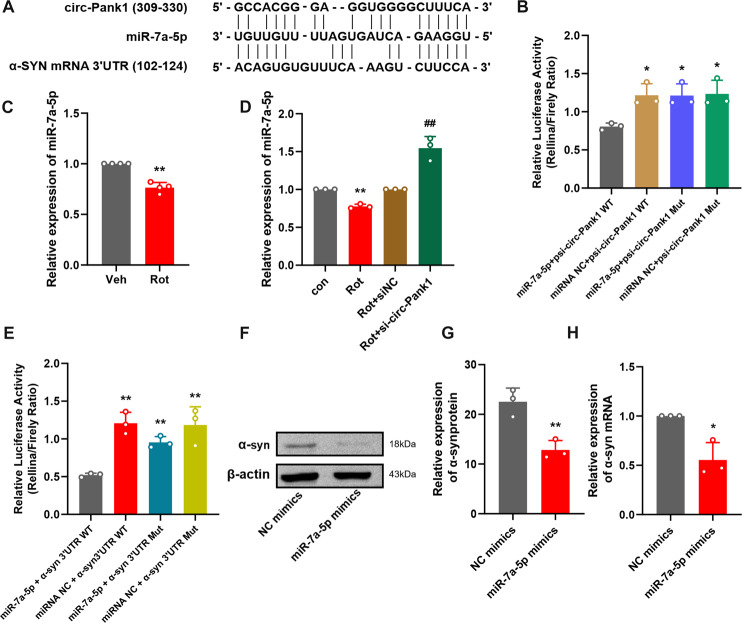
Interaction between circ-Pank1/miR-7a-5p/a-syn in MN9D cells. **A** Schematic diagram of binding sites between circ-Pank1 and the α-syn mRNA 3’UTR and miR-7a-5p. **B** The regulatory effect of circ-Pank1 on the expression of miR-7a-5p was verified by dual-luciferase reporter gene detection. The psi-circ-Pank-WT/Mut plasmid and miR-7a-5p mimic were cotransfected into HEK293T cells, and the luciferase activity was measured after 24 h (*n* = 3). **C** qRT–PCR was used to detect the expression of miR-7a-5p in the SN of PD model mice treated with rotenone (*n* = 4). **D** After si-circ-Pank1 and the corresponding control were transferred to the MN9D cell model treated with rotenone, the expression changes of miR-7a-5p were detected by qRT–PCR (*n* = 3). **E** The regulatory effect of miR-7a-5p on α-syn expression was verified by the dual-luciferase reporter gene detection. The psi-α-syn WT/Mut plasmid and miR-7a-5p mimic were co-transfected into HEK293T cells. After 24 h, the luciferase activity was measured. **F**–**H** After the transfection of miR-7a-5p mimics in MN9D cells (*n* = 3), Western blot analysis of α-syn protein levels **F**, **G** qRT–PCR detection of α-syn mRNA expression **H**. Data are presented as mean ± SD. *P < 0.05, **P < 0.01.

As circRNAs in the cytoplasm mostly function as miRNA sponges to regulate downstream genes, followingly, we explored the ability of circ-Pank1 to bind to miRNAs [21]. Online bioinformatics databases (http://www.targetscan.org/vert_80/) were used to identify potential miRNAs that targeted α-syn mRNA sponged by circ-Pank1, and miR-7a-5p was predicted. Moreover, we identified the conserved binding sites for miR-7a-5p on circ-Pank1 and the 3′UTR of α-syn (Fig. 4A). We further studied the relationship between circ-Pank1 and miR-7a-5p. Luciferase reporter plasmids with a wild-type sequence of circ-Pank1 (psi-circ-Pank1 WT) or mutant sequence (psi-circ-Pank1 Mut) in the binding sites of miR-7a-5p were generated. As shown in Fig. 4B, the miR-7a-5p mimic transfection suppressed the luciferase activity of the WT plasmid. Meanwhile, the suppressive effect of miR-7a-5p mimic transfections was significantly abolished in the mutant plasmid. Furthermore, the qRT–PCR results show that the miR-7a-5p expression significantly decreased in samples of the SN from mouse models of PD (Fig. 4C) and the circ-Pank1 knockdown increased the expression level of miR-7a-5p in MN9D cells treated with rotenone (Fig. 4D).

### MiR-7a-5p targets the expression of α-syn

According to TargetScan and microRNA.org, the web-based prediction software for targets of miRNAs shows that the α-syn 3′UTR region contains the conserved MRE of miR-7a-5p (Fig. 4A). To evaluate whether α-syn is the direct target of miR-7a-5p, luciferase reporter plasmids with the wild-type α-syn mRNA 3′UTR sequence (α-syn 3′UTR WT) and mutant α-syn mRNA 3′UTR sequence (α-syn 3′UTR Mut) were generated. The results reveal that the transfection of miR-7a-5p mimics significantly reduced the luciferase activity of α-syn 3′UTR WT but not that of α-syn α-syn 3′UTR Mut in MN9D cells (Fig. 4E). Western blot analysis shows that the transfection of miR-7a-5p mimics significantly reduced α-syn protein and mRNA levels in vitro (Fig. 4F–H). These results indicate that α-syn was the direct target of miR-7a-5p.

### Circ-Pank1 regulates dopaminergic neuron injury through targeted regulation of miR-7a-5p/α-syn

**Fig. 5:**
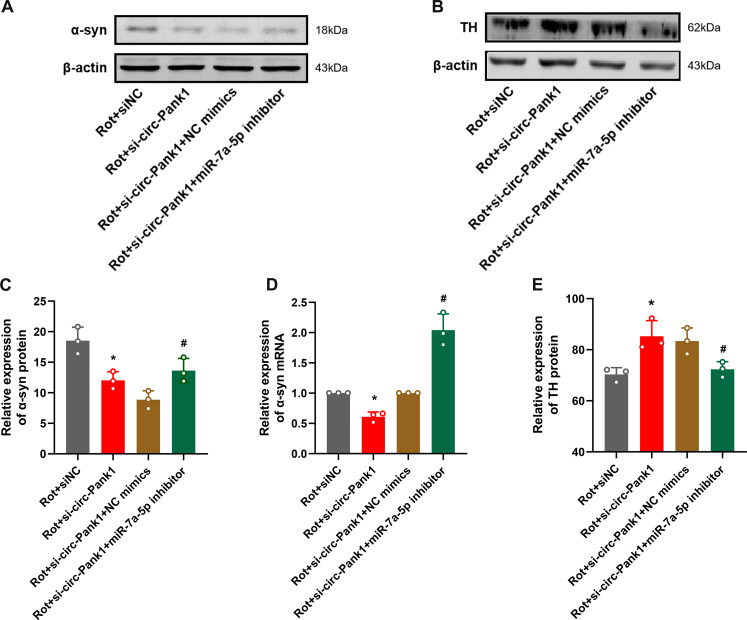
Circ-Pank1 regulates the dopaminergic neuron damage by targeting miR-7a-5p/α-syn. After si-circ-Pank1 and miR-7a-5p inhibitors and corresponding controls were transferred to the MN9D cell model treated with rotenone (*n* = 3), Western blot analysis of α-syn and TH protein levels (**A**–**C**, **E**). qRT–PCR detects the level of α-syn mRNA (**D**). Data are presented as mean ± SD. *P < 0.05, **P < 0.01.

To further confirm that the regulation of dopaminergic neuron injury by circ-Pank1 depends on miR-7a-5p-mediated α-syn levels, rescue experiments were conducted by co-transfecting si-circ-Pank1 and miR-7a-5p inhibitors into MN9D cells treated with rotenone. We performed the following Western blot and qRT–PCR experiments. The miR-7a-5p inhibitor partially abolished the effect of si-circ-Pank1 on the expression of α-syn (Fig. 5A, C, D). Notably, dopaminergic neuron injury had a similar change pattern to α-syn. Immunohistochemical staining and Western blot analysis show that the co-transfection of miR-7a-5p inhibitor significantly abolished the effect of si-circ-Pank1 on the upregulation of TH-positive neurons in rotenone treated MN9D cells (Fig. 5B, E). Collectively, these data demonstrate that circ-Pank1 promotes dopaminergic neuron injury by directly targeting the miR-7a-5p/α-syn signaling.

### Silencing circ-Pank1 in vivo can inhibit the damage of dopaminergic neurons caused by Rotenone treatment

**Fig. 6:**
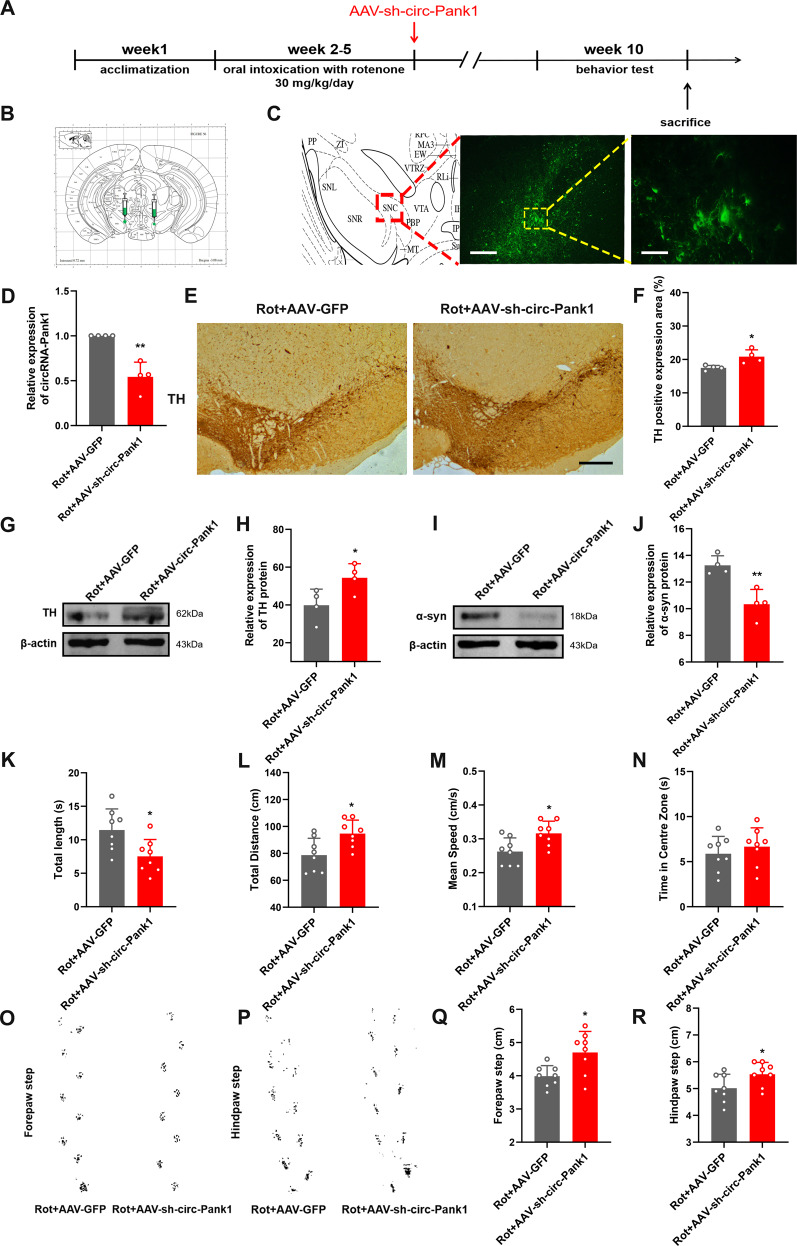
Silencing circ-Pank1 can improve the PD-like motor dysfunction in mice treated with rotenone. **A** Experimental schematic diagram. After 4 weeks of continuous administration of rotenone, the adeno-associated virus vector (AAV-sh-circ-Pank1) with fluorescence-specific knockdown of circ-Pank1 (AAV-sh-circ-Pank1) and the control GFP were injected by stereotactic brain injection. Adeno-associated virus vector (AAV-GFP) was injected into the SN of PD model mice treated with rotenone. **B** Schematic diagram of the brain stereotactic injection site of virus. **C** Representative fluorescence image of the virus-transfected section; scale bars, 100 μm and 50 μm. **D** qRT–PCR was used to detect the expression of circ-Pank1 after injection of AAV-sh-circ-Pank1 into the SN of PD model mice treated with rotenone (*n* = 4). **E**, **F** Immunohistochemical detection of the number of TH-positive cells after the injection of AAV-sh-circ-Pank1 into PD model mice treated with rotenone; scale bars, 500 μm (*n* = 4). **G**, **H** Western blot analysis of α-syn protein levels in the SN of PD model mice treated with rotenone after injection of AAV-sh-circ-Pank1. **I**, **J** Western blot analysis of TH protein levels in the SN of PD model mice treated with rotenone after the injection of AAV-sh-circ-Pank1 (*n* = 4). **K**–**R** PD model mice treated with rotenone after the injection of AAV-sh-circ-Pank1 were subjected to a pole climbing test **K**, field test (**L**–**N**) and step measurement (**O**–**R**) to evaluate the exercise ability (*n* = 8). Data are presented as mean ± SD. *P < 0.05, **P < 0.01.

To more comprehensively confirm the protective effect of si-circ-Pank1 in the PD model treated with rotenone, we investigated the effects of circ-Pank1 knockdown on the PD-like pathology and motor phenotype in vivo. PD model mice treated with rotenone received a stereotactic injection of AAV-sh-circ-Pank1 into both sides of the SN. First, we confirmed successful transfection at the site of the SN by tracing fluorescence-labelled AAV-sh-circ-Pank1 (Fig. 6A–C). The qRT–PCR assays confirm that circ-Pank1 significantly decreased following the AAV-sh-circ-Pank1 injection (Fig. 6D). To explore the effects of circ-Pank1 knockdown on dopaminergic neurons, Western blot analysis showed that AAV-sh-circ-Pank1 injection significantly reduced the expression of a-syn in SN (Fig. 6I, J) and increased TH-positive neurons in the SN (Fig. 6E–H).

In addition, AAV-sh-circ-Pank1 attenuated locomotor dysfunction after treatment with rotenone. In the pole experiment, the Rot+AAV-sh-circ-Pank1 group took less time to climb down the pole than the Rot+AAV-GFP group (Fig. 6K). In the open field test, there was no significant change in residence time in the central area between the Rot+AAV-GFP group and Rot+AAV-sh-circ-Pank1 group (Fig. 6L). The Rot+AAV-sh-circ-Pank1 group had significantly higher total distance travelled and average speed than the Rot+AAV-GFP group (Fig. 6M, N). The step measurement shows that the forepaw step and hindpaw step distance of the Rot+AAV-sh-circ-Pank1 group significantly increased (Fig. 6O–R). The stereotactic injection of AAV-sh-circ-Pank1 into the SN significantly increased the number of TH-positive neurons, motor activity, step distance, and balance in vivo. Taken together, these findings demonstrate that circ-Pank1 knockdown ameliorates the dopaminergic neuron loss and impaired locomotor behaviors.

## Discussion

**Fig. 7:**
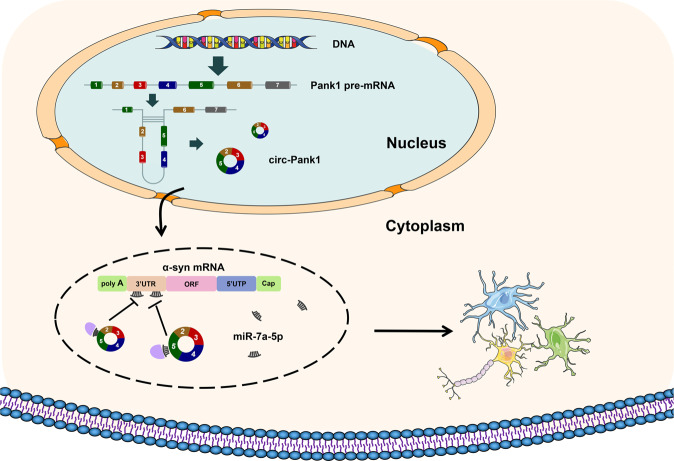
Schematic diagram, circ-Pank1 acts as a competitive endogenous RNA (ceRNA) and regulates the expression of α-syn by adsorbing miR-7a-5p to affect the damage of dopaminergic neurons.

In this study, we identified that circ-Pank1, which is an Nrf2-associated circRNA, was highly expressed in PD model mice treated with rotenone. The inhibition of circ-Pank1 expression ameliorates the dopaminergic neuron injury and locomotor dysfunction. Further research reveals that circ-Pank1 acts as a competing endogenous RNA (ceRNA) and regulates the α-syn expression by adsorbing miR-7a-5p. Blocking the circ-Pank1/miR-7a-5p/α-syn axis effectively rescues the locomotor dysfunction in PD model mice (Fig. 7).

In PD, disease symptoms will not appear until the dopaminergic neurons in the SN are damaged by more than 60% [22], which requires seeking new treatments and prevention strategies to delay the onset and slow down the deterioration of the disease. Many studies have shown that noncoding RNAs play important roles in neurodegenerative diseases and aging-related diseases. Compared with other linear RNAs (mRNAs, miRNAs, or lncRNAs), circRNAs are abundantly, conservatively, and stably expressed in the brain. Therefore, it is an important indicator of changes in response to certain diseases and a rising star as a therapeutic target. Much evidence shows that circRNAs play a pivotal role in PD [23]. Hanan et al. studied the expression of circRNAs in three different brain regions of PD patients, and they identified 24 circRNAs that were differentially expressed in the three brain regions. Among them, the expression of circSLC8A1 most significantly increases and is related to the pathological changes of PD. As an important antioxidant transcription factor, Nrf2 plays a key role in PD [24]. Specifically, in PD animal models, the inactivation of Nrf2 can aggravate nerve damage, and its overexpression can reduce neurodegeneration and prevent synucleinopathy [25]. According to previous studies, Nrf2 can participate in the occurrence of diseases by regulating the transcription of noncoding RNAs [18, 19]. In our previous work, we found that circRNAs were differentially expressed in the SN of Nrf2^+/+^ and Nrf2^-/-^ mice [20]. Till now, we have screened and verified the 2 upregulated and 2 downregulated circRNAs with a large multiple and a small *P* value in the PD model. The expression of mmu-circRNA-32463 was most obvious, and it was derived from the Pank1 gene, thus we named it circ-Pank1.

In our study, we found that the expression of circ-Pank1 was significantly upregulated in the SN of PD model mice treated with rotenone. The results show that the upregulation of circ-Pank1 is related to the damage to dopaminergic neurons in vivo and in vitro. PD is caused by the specific loss of TH-positive dopaminergic neurons present in the SN. We found that downregulation of circ-Pank1 in vitro could ameliorate neuronal injury induced by rotenone.

The aggregation and misfolding of α-syn is an important part of Lewy bodies and the most important pathogenic factor for PD [26]. α-syn induces disorder of the dopamine system by affecting the expression of TH and subsequently makes PD occur [27]. In a mouse model, the α-syn overexpression significantly reduced the TH activity [28]. Our results show that the expression of α-syn can be reduced by the knockdown of circ-Pank1. However, the mechanism by which circ-Pank1 regulates the expression of α-syn remains unclear.

We found that circ-Pank1 was mainly located in the cytoplasm. Most of the studies on circRNAs have focused on the mechanism by which circRNAs function as sponges of miRNAs through interactions with the miRNA-Ago2 complex, which change the expression of the target genes [14, 21, 29, 30]. However, this ceRNA effect depends on the cytoplasmic localization of circRNA. In addition to miRNAs, circRNAs can bind to RBPs and sequester them from their natural targets or regulate their activity/stability [31–34]. CircRNAs localized in the nucleus can function as modulators of transcription of their host genes [35]. Meanwhile, circRNAs have coding functions. Some circRNAs can be translated by ribosomes and encode polypeptides to perform regulatory functions [36]. CircRNA ciRS-7 leads to an increase in miR-7 and a decrease in its target mRNA, which affects the brain function [14, 30]. α-syn is one of the targets of miR-7, and studies have confirmed that miR-7 can regulate the expression of α-syn [37]. In the present study, circ-Pank1 was predominantly localized to the cytoplasm and acted as a sponge to competitively bind with miR-7a-5p, as demonstrated through dual-luciferase reporter assays. Furthermore, α-syn was predicted as the target of miR-7a-5p by several online databases. Here, the interaction between miR-7a-5p and α-syn was confirmed in MN9D cells. Moreover, we observed that the miR-7a-5p expression level decreased in Rot-treated MN9D cells and the α-syn protein level was affected by transfection with the miR-7a-5p inhibitor, which supports the hypothesis that miR-7a-5p targets α-syn.

In conclusion, this study indicates that circ-Pank1 is highly expressed and promotes dopaminergic neuron damage by regulating the α-syn expression by sponging miR-7a-5p. These findings reveal a novel signaling pathway that may provide new ideas to prevent and diagnose PD.

There are several limitations of this study, which should be acknowledged. First, PD model mice treated with rotenone do not represent real PD patients. We did not examine the expression of circ-Pank1 in PD human patient samples, neither the therapeutic effect and impact of circ-Pank1/miR-7a-5p/α-syn axis in human patients. The therapeutic effect and impact in human patients will be more meaningful. Secondly, as a circular RNA, there are many miRNA binding sites on its surface. We only selected one of them for verification. Maybe there are other mechanisms of circ-Pank1 that we have not discovered yet. In the future, we will pay more attention to other regulatory mechanisms of circ-Pank1 and the role of circRNAs in the clinical treatment of PD patients, to make our research more accurate and meaningful.

## Materials and Methods

### Rotenone mouse model and drug administration

All animal experiments were approved by the Ethics Committee of Hebei Medical University and strictly complied with the National Institutes of Health Guidelines for the Care and Use of Laboratory Animals. ICR mice (aged 10–12 weeks) were purchased from Charles River and kept under constant temperature (21–22 °C) with free access to food and water in a 12-hour light/dark cycle. Rotenone (APExBIO, Houston, USA) was administered orally once daily by intragastric gavage at a dose of 30 mg/kg for 28 days. Rotenone was suspended in 0.5% carboxymethyl cellulose sodium salt (CMC) and administered orally once daily at a concentration of 6 mL/kg body weight. CMC (0.5%) was administered orally as a vehicle to the control mice.

### Behavior analysis

Open field. The field test was used to determine the spontaneous exploratory activity of the mice. The mice were put into the testing room and habituated the day before the experiment. The mice were placed in the center of the open field, and their movement was monitored (videotaped) for 5 min. Videos were analyzed in terms of the following parameters: central area time, total distance, and average speed.

Pole climbing. We made a straight wooden pole with a diameter of 0.8 cm and a height of 60 cm. There was a small wooden ball on the top of the pole, and gauze was applied to prevent the mice from slipping. A mouse was placed on a vertical wooden pole with its head facing upwards, and the time it took to climb to the bottom of the pole was measured. After 5 days of training, the mice smoothly climbed to the bottom. The experiment officially started on the 6th day, each test interval was more than 3 min, and the average value was taken 3 times.

Footprint test. The mouse was encouraged to walk in a straight line (a narrow corridor) for 3 days. On the 4th day, the fore and hind paws were painted with dyes of different colors, and the mouse was encouraged to walk on absorbent paper. The footprint patterns are subsequently analyzed for a range of measurements.

### Immunohistochemistry

The mice were sacrificed using sodium pentobarbital (60 mg/kg) and perfused with 4% paraformaldehyde (PFA). Subsequently, the brains were extracted and post-fixed in 4% PFA, sunk through a 30% sucrose solution, and sectioned (16 μm) in the coronal plane on a freezing sliding microtome. The brain slices were incubated with primary antibodies, including rabbit antibodies to anti-TH (Servicebio, Wuhan, China, GB11181) and oligomer A11 polyclonal antibody (Invitrogen, Shanghai, China, AHB0052), overnight at 4 °C. The sections were washed with PBS. Biotinylated secondary antibody was added and incubated for 2 h; then, the color was developed with 3,3’-diaminobenzidine tetrachloride (DAB) (ORIGENE, Wuxi, China).

### Western blotting

After repeated administration for 28 days, the SN in treated mice or MN9D cells was rapidly removed and homogenized with RIPA buffer (Solarbio, Beijing, China) supplemented with 1 mM phenylmethylsulfonyl fluoride (PMSF). A BCA assay (Solarbio, Beijing, China) was used to quantify the protein concentrations. The protein samples (40 μg) were electrophoresed in sodium dodecyl sulfate-polyacrylamide gels (SDS–PAGE) and transferred onto polyvinylidene difluoride membranes (PVDF). The resulting blots were blocked with 5% nonfat skimmed milk in TBS for 1 h at room temperature. Then, the membranes were incubated with the primary antibodies anti-TH (Servicebio, Wuhan, China, GB11181), anti-α-syn (Genetex, CA, USA, TX133367), and β-actin (ABclonal, Wuhan, China, AC026) at 4 °C overnight. Then, the membranes were incubated with anti-rabbit horseradish peroxidase (HRP)-conjugated secondary antibodies (Rockland, Beijing, China) for 2 h at room temperature. All immunoblot images were taken using the Odyssey infrared laser scanning imaging system. A relative densitometry analysis of Western blots was performed using the ImageJ Program.

### Real-Time qRT–PCR

Total RNA was extracted from the SN in treated mice or MN9D cells using TRIzol reagent (ZOMANBIO, Beijing, China) and inversely transcribed into cDNA using the HiScript III RT SuperMix for qPCR (+gDNA wiper) (Vazyme, Nanjing, China). Quantitative real-time PCR was performed to measure circ-Pank1, miR-7a-5p, and SNCA expression using the ChamQ Universal SYBR qPCR Master Mix (Vazyme, Nanjing, China). Relative expression levels were calculated using the 2^-ΔΔCT^ method, and primer sequences (5′−3′) were as follows:

mmu_circRNA_Pank1

Forward: 5′ AAGAAAAGAGAGAGTCCATCAGCAA 3′

Reverse: 5′ TTCAAAGTAAACCAGCTTAACCAGG 3′

mmu_circRNA_34132

Forward: 5′ TGAAGAACTCTGTCTACCGAAGCC 3′

Reverse: 5′ TAGTCAAAGCCTTGCACGGGAT 3′

mmu_circRNA_34106

Forward: 5′ GGCTGCTGAAGAGTGAACTTGGAT 3′

Reverse: 5′ AGGTGAGGATGGAGCTGTGTCTTC 3′

mmu_circRNA_015216

Forward: 5′ AAAGTCAGATGTGTGGTCATTTGGAA 3′

Reverse: 5′ TTCCACTCCATAATGACTGGCACTT 3′

β-actin

Forward: 5′ TCATCACTATTGGCAACGAGCGGT 3′

Reverse: 5′ GTGTTGGCATAGAGGTCTTTACG 3′

SNCA

Forward: 5′ GGGAGTCCTCTATGTAGGTTCC 3′

Reverse: 5′ TCCAACATTTGTCACTTGCTCT 3′

miR-7a-5p and U6 From TIANGEN Company

### In Situ Hybridization

An appropriate amount of cells was inoculated into a 24-well plate. We fixed them with 4% paraformaldehyde at room temperature for 10 min, washed with PBS for 5 min, added 1 ml permeabilization solution to each well 3 times, let stand at 4 °C for 5 min, and washed with PBS for 5 min, 3 times. We added 200 μL of the prehybridization solution to each well, blocked at 37 °C for 30 min, and added 2.5 µM circRNA FISH Probe Mix and FISH Probe Mix to 100 µl preheated hybridization solution under dark conditions. Then, the prehybridization solution was discarded from each well, 100 μL of the probe-containing hybridization solution was added, and the plate was kept away from light overnight at 37 °C. Hybridization lotions I, II, and III were preheated to 42 °C and the cells were washed with hybridization wash I in the dark 3 times for 5 min each time. The cells were washed with mixed lotion II and mixed lotion III for 5 min, each time under dark conditions, and subsequently washed with PBS for 5 min at room temperature. DAPI staining was conducted for 10 min under dark conditions. The cells were washed 3 times with PBS for 5 min each time. Under dark conditions, the cell slide was removed and fixed on a glass slide with a mounting tablet, and fluorescence detection was performed with a confocal microscope (the maximum excitation light length was 555 nm and the maximum emission wavelength was 570 nm).

### Cell culture and treatment

MN9D mouse dopaminergic neurons were cultured in DMEM (Procell, Wuhan, China) supplemented with 10% FBS (CLARK, Virginia, U.S.) in a 37 °C incubator with 5% CO_2_. To establish a cellular PD model in vitro, MN9D cells were treated with 1 µM rotenone for 24 h.

To downregulate Nrf2 and circ-Pank1, small interfering RNAs targeting Nrf2 (si-Nrf2) and circ-Pank1 (si-circ-Pank1) were synthesized by Gemma. To upregulate miR-7a-5p, targeting miR-7a-5p mimics were synthesized by Gemma. To downregulate miR-7a-5p, the company synthesized inhibitors targeting miR-7a-5p inhibitors. When the cells reached 70–80% confluence, 40 nmol si-Nrf2, si-circ-Pank1, miR-7a-5p mimics, miR-7a-5p inhibitors, and their corresponding negative controls were transfected with Lipofectamine 3000 (Thermo Fisher Scientific, Waltham, USA).

### Luciferase reporter assay

Based on the putative miR-7a-5p binding sites in circ-Pank1 (circ-Pank1-WT) and the α-syn 3’UTR (α-syn-WT), circ-Pank1 and the α-syn 3’UTR with mutated miR-7a-5p binding sites (circ-Pank1-Mut, α-syn-Mut) were generated. A luciferase reporter of psi-CHECK2 vectors containing mutated sites was constructed by General Biosystems. psi-circ-Pank-WT/Mut or psi-α-syn WT/Mut was cotransfected with miR-7a-5p mimic into HEK293T cells using Lipofectamine 3000 Transfection Reagent. Luciferase activity was measured 24 h after transfection using the Dual-Luciferase® Reporter Assay System (Promega).

### Cell viability analysis

According to the manufacturer’s instructions, the cell viability was measured by Cell Counting Kit-8 (CCK-8) (APExBIO, Houston, USA). MN9D cells were seeded in 96-well plates and cultured overnight. After different treatments, CCK-8 solution (100 μl/well) was added to each well and incubated at 37 °C for 4 h. A microplate reader was used to measure the absorbance of each well at 450 nm.

### Adeno associated virus injection

The mice anaesthetized with pentobarbital (60 mg/kg) were intracerebrally injected with the circRNA-Pank1 adeno-associated virus vector AAV-sh-circRNA-Pank1 or AAV-GFP (from Hanbio Biotechnology Shanghai) into the SN pars compacta SNpc (AP: −3.0, ML: + 1.1, DV: −4.5). This procedure was conducted using a 1-μl Hamilton syringe with a 33-gauge tip needle, 0.5 μl/side at a rate of 0.1 μl/min for 5 min.

### Statistical analysis

All in vitro experiments were performed in triplicate and each determination was repeated 3 times. The average of 3 repeated measurements was used for statistical analysis. All analyses were conducted by the SPSS 17.0 software. The data are presented as the means ± standard deviations (SD). A two-tailed Student’s t-test was used to analyze the comparison between 2 groups. Statistical significance between the groups is represented as *P < 0.05, **P < 0.01.

## Supplementary information

checklist

Original Data File For Image

Original Data File For Western Blots

## Data Availability

The datasets used and/or analyzed during the current study are available from the corresponding author on reasonable request.

## References

[1] Spillantini MG, Crowther RA, Jakes R, Hasegawa M, Goedert M (1998). alpha-Synuclein in filamentous inclusions of Lewy bodies from Parkinson’s disease and dementia with lewy bodies. Proc Natl Acad Sci USA.

[2] Tarakad A, Jankovic J (2017). Diagnosis and Management of Parkinson’s Disease. Semin Neurol.

[3] Dickson DW (2012). Parkinson’s disease and parkinsonism: neuropathology. Cold Spring Harb Perspect Med.

[4] Dickson DW (2018). Neuropathology of Parkinson disease. Parkinsonism Relat Disord.

[5] Conn SJ, Pillman KA, Toubia J, Conn VM, Salmanidis M, Phillips CA (2015). The RNA binding protein quaking regulates formation of circRNAs. Cell.

[6] Sibley CR, Blazquez L, Ule J (2016). Lessons from non-canonical splicing. Nat Rev Genet.

[7] Zhang XO, Dong R, Zhang Y, Zhang JL, Luo Z, Zhang J (2016). Diverse alternative back-splicing and alternative splicing landscape of circular RNAs. Genome Res.

[8] Rybak-Wolf A, Stottmeister C, Glažar P, Jens M, Pino N, Giusti S (2015). Circular RNAs in the Mammalian Brain Are Highly Abundant, Conserved, and Dynamically Expressed. Mol Cell.

[9] You X, Vlatkovic I, Babic A, Will T, Epstein I, Tushev G (2015). Neural circular RNAs are derived from synaptic genes and regulated by development and plasticity. Nat Neurosci.

[10] Gruner H, Cortés-López M, Cooper DA, Bauer M, Miura P (2016). CircRNA accumulation in the aging mouse brain. Sci Rep.

[11] Liu EY, Cali CP, Lee EB (2017). RNA metabolism in neurodegenerative disease. Dis Models Mech.

[12] Chen LL (2016). The biogenesis and emerging roles of circular RNAs. Nat Rev Mol Cell Biol.

[13] Jeck WR, Sharpless NE (2014). Detecting and characterizing circular RNAs. Nat Biotechnol.

[14] Hansen TB, Jensen TI, Clausen BH, Bramsen JB, Finsen B, Damgaard CK (2013). Natural RNA circles function as efficient microRNA sponges. Nature..

[15] Lastres-Becker I, Ulusoy A, Innamorato NG, Sahin G, Rábano A, Kirik D (2012). α-Synuclein expression and Nrf2 deficiency cooperate to aggravate protein aggregation, neuronal death and inflammation in early-stage Parkinson’s disease. Hum Mol Genet.

[16] Gan L, Johnson JA (2014). Oxidative damage and the Nrf2-ARE pathway in neurodegenerative diseases. Biochimica et biophysica acta.

[17] Johnson JA, Johnson DA, Kraft AD, Calkins MJ, Jakel RJ, Vargas MR (2008). The Nrf2-ARE pathway: an indicator and modulator of oxidative stress in neurodegeneration. Ann N. Y Acad Sci.

[18] Gao M, Zhao B, Chen M, Liu Y, Xu M, Wang Z (2017). Nrf-2-driven long noncoding RNA ODRUL contributes to modulating silver nanoparticle-induced effects on erythroid cells. Biomaterials..

[19] Thai P, Statt S, Chen CH, Liang E, Campbell C, Wu R (2013). Characterization of a novel long noncoding RNA, SCAL1, induced by cigarette smoke and elevated in lung cancer cell lines. Am J Respir Cell Mol Biol.

[20] Yang JH, Zhang RJ, Lin JJ, Cao MC, Wang Q, Cui HX (2018). The Differentially Expressed Circular RNAs in the Substantia Nigra and Corpus Striatum of Nrf2-Knockout Mice. Cell Physiol Biochem: Int J Exp Cell Physiol, Biochem, Pharmacol.

[21] Zheng Q, Bao C, Guo W, Li S, Chen J, Chen B (2016). Circular RNA profiling reveals an abundant circHIPK3 that regulates cell growth by sponging multiple miRNAs. Nat Commun.

[22] Kalia LV, Lang AE (2015). Parkinson’s disease. Lancet (Lond, Engl).

[23] Hanan M, Simchovitz A, Yayon N, Vaknine S, Cohen-Fultheim R, Karmon M (2020). A Parkinson’s disease CircRNAs Resource reveals a link between circSLC8A1 and oxidative stress. EMBO Mol Med.

[24] Anandhan A, Nguyen N, Syal A, Dreher LA, Dodson M, Zhang DD (2021). NRF2 Loss Accentuates Parkinsonian Pathology and Behavioral Dysfunction in Human α-Synuclein Overexpressing Mice. Aging Dis.

[25] Kumar H, Koppula S, Kim IS, More SV, Kim BW, Choi DK (2012). Nuclear factor erythroid 2-related factor 2 signaling in Parkinson disease: a promising multi therapeutic target against oxidative stress, neuroinflammation and cell death. CNS Neurol Disord Drug Targets.

[26] Bridi JC, Hirth F (2018). Mechanisms of α-Synuclein Induced Synaptopathy in Parkinson’s Disease. Front Neurosci.

[27] Daubner SC, Le T, Wang S (2011). Tyrosine hydroxylase and regulation of dopamine synthesis. Arch Biochem biophysics.

[28] Masliah E, Rockenstein E, Veinbergs I, Mallory M, Hashimoto M, Takeda A (2000). Dopaminergic loss and inclusion body formation in alpha-synuclein mice: implications for neurodegenerative disorders. Sci (N. Y, NY).

[29] Bach DH, Lee SK, Sood AK (2019). Circular RNAs in Cancer. Molecular therapy. Nucleic Acids.

[30] Memczak S, Jens M, Elefsinioti A, Torti F, Krueger J, Rybak A (2013). Circular RNAs are a large class of animal RNAs with regulatory potency. Nature..

[31] Ashwal-Fluss R, Meyer M, Pamudurti NR, Ivanov A, Bartok O, Hanan M (2014). circRNA biogenesis competes with pre-mRNA splicing. Mol Cell.

[32] Du WW, Fang L, Yang W, Wu N, Awan FM, Yang Z (2017). Induction of tumor apoptosis through a circular RNA enhancing Foxo3 activity. Cell Death Differ.

[33] Liu CX, Li X, Nan F, Jiang S, Gao X, Guo SK (2019). Structure and Degradation of Circular RNAs Regulate PKR Activation in Innate Immunity. Cell..

[34] Rossi F, Legnini I, Megiorni F, Colantoni A, Santini T, Morlando M (2019). Circ-ZNF609 regulates G1-S progression in rhabdomyosarcoma. Oncogene..

[35] Li Z, Huang C, Bao C, Chen L, Lin M, Wang X (2015). Exon-intron circular RNAs regulate transcription in the nucleus. Nat Struct Mol Biol.

[36] Li X, Yang L, Chen LL (2018). The Biogenesis, Functions, and Challenges of Circular RNAs. Mol Cell.

[37] Lu D, Xu AD (2016). Mini Review: Circular RNAs as Potential Clinical Biomarkers for Disorders in the Central Nervous System. Front Genet.

