# Supplementary material for: circ-Pank1 promotes dopaminergic neuron neurodegeneration through modulating miR-7a-5p/α-syn pathway in Parkinson’s disease
Source: Cell Death Dis. 2022 May 19;13(5):477. doi: 10.1038/s41419-022-04934-2 (PMC9120029; doi:10.1038/s41419-022-04934-2)
Supplement: Supplementary file 2 — Original Data File For Image [file 41419_2022_4934_MOESM2_ESM.pdf]

Fig1. A

Veh Rot

TH

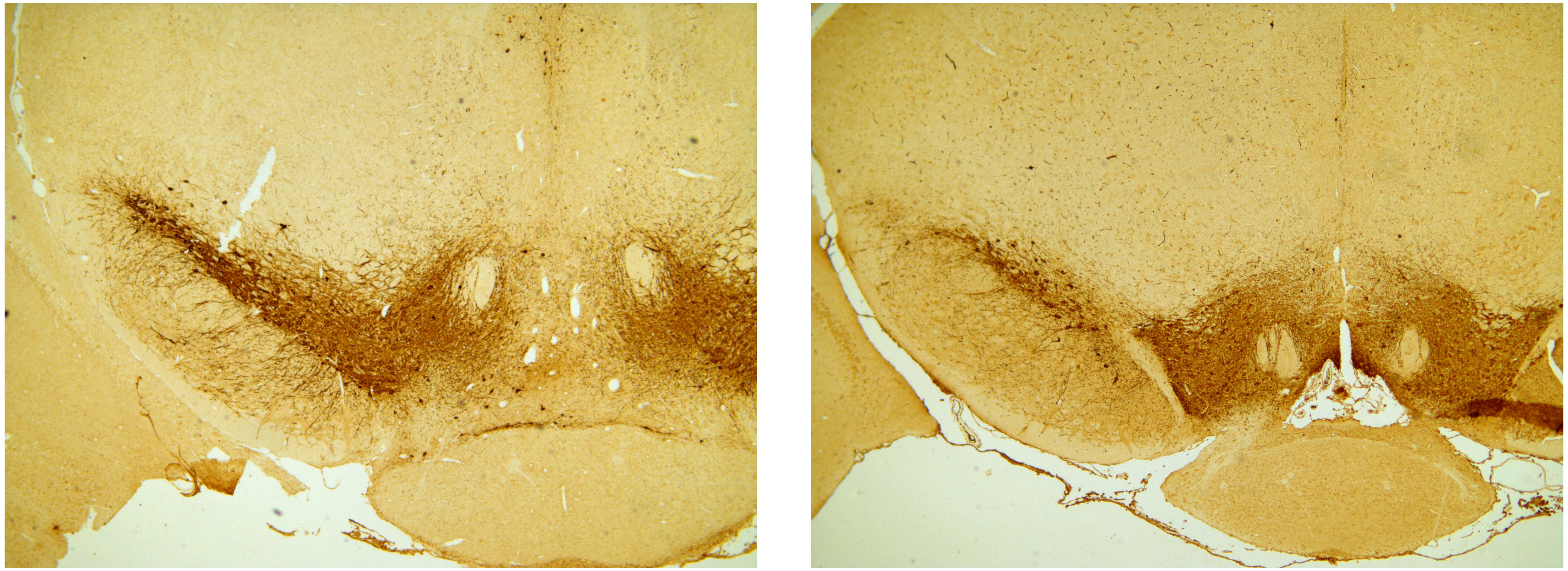

The Original image of TH immunohistochemical stainingis in Fig1. A

Figure2.C

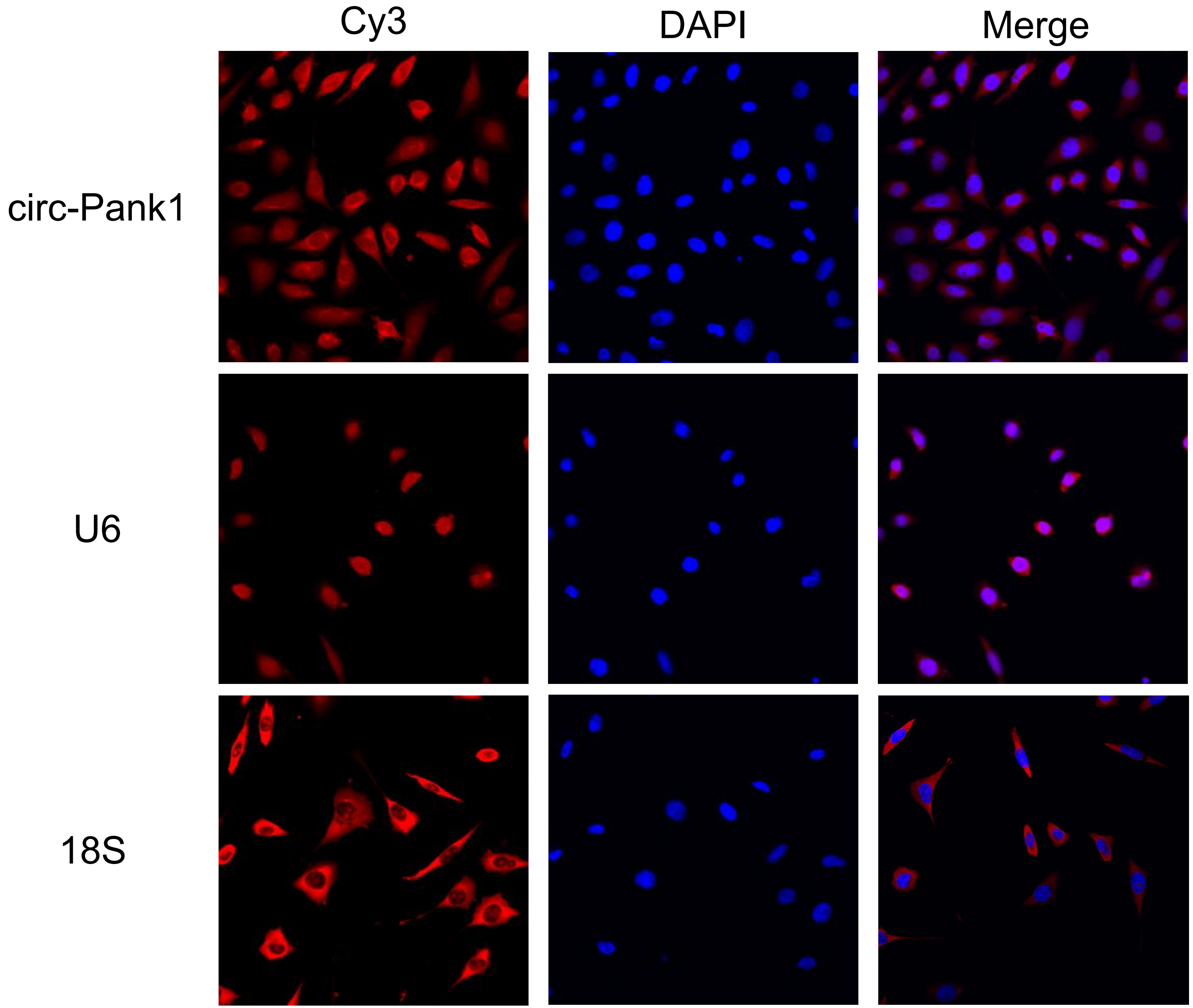

The original image of circ-Pank1, nuclear control U6 and cytoplasmic control 18S fluorescence in situ hybridization in Figure2.C

Fig6. C

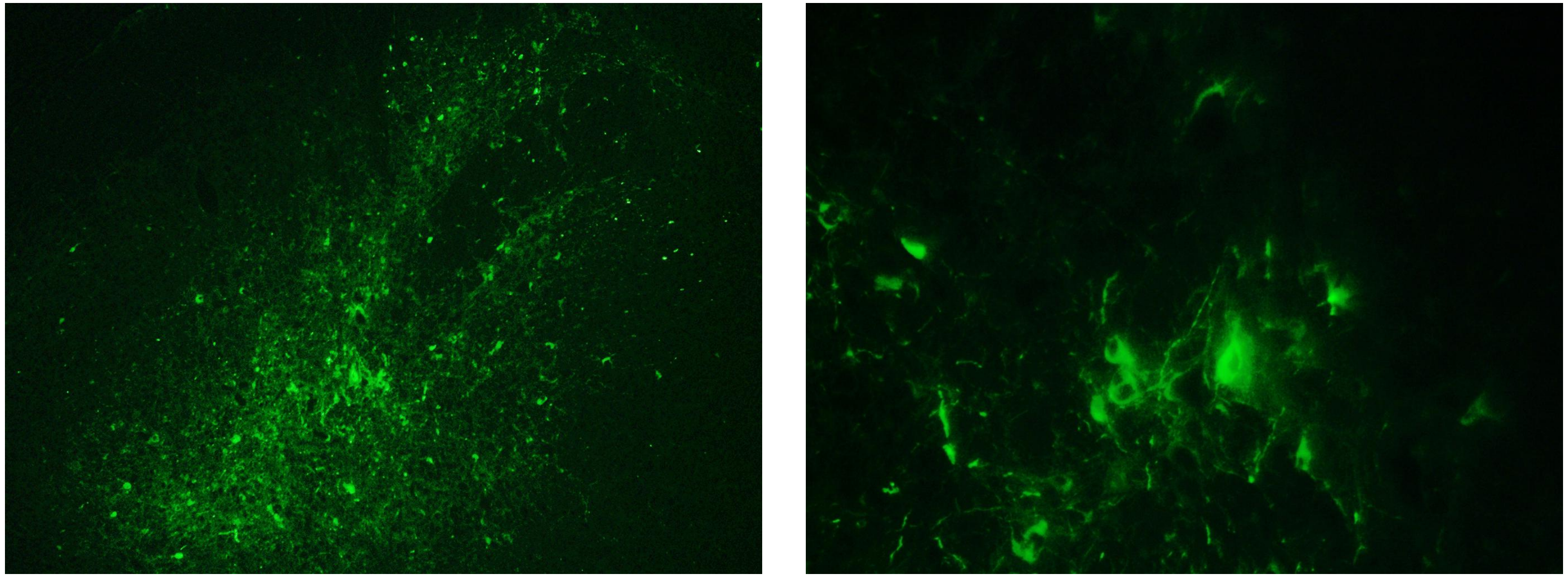

Representative fluorescence image of the virus-transfected section in Fig6.C

Fig6. E

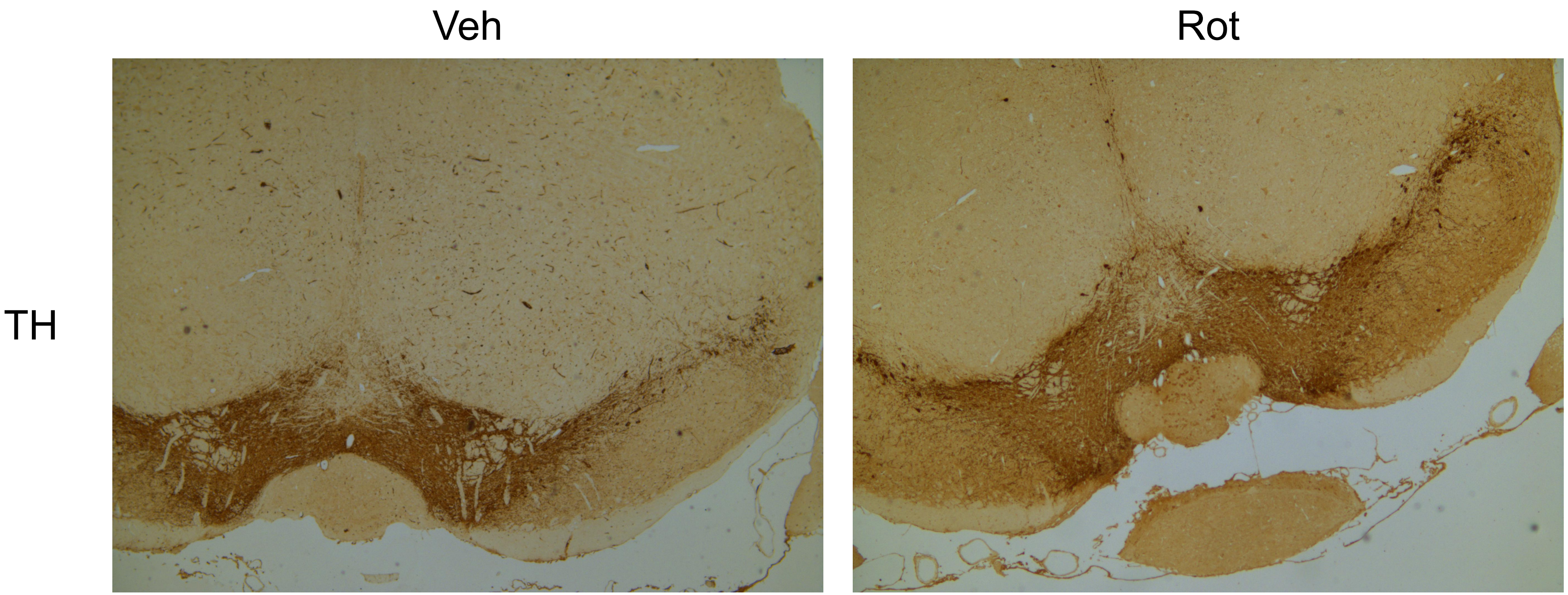

The Original image of TH immunohistochemical stainingis in Fig6. E
