# Supplementary material for: circ-Pank1 promotes dopaminergic neuron neurodegeneration through modulating miR-7a-5p/α-syn pathway in Parkinson’s disease
Source: Cell Death Dis. 2022 May 19;13(5):477. doi: 10.1038/s41419-022-04934-2 (PMC9120029; doi:10.1038/s41419-022-04934-2)
Supplement: Supplementary file 3 — Original Data File For Western Blots [file 41419_2022_4934_MOESM3_ESM.pdf]

Tailor the membrane to incubate different antibodies

Fig1. C   Fig3. A

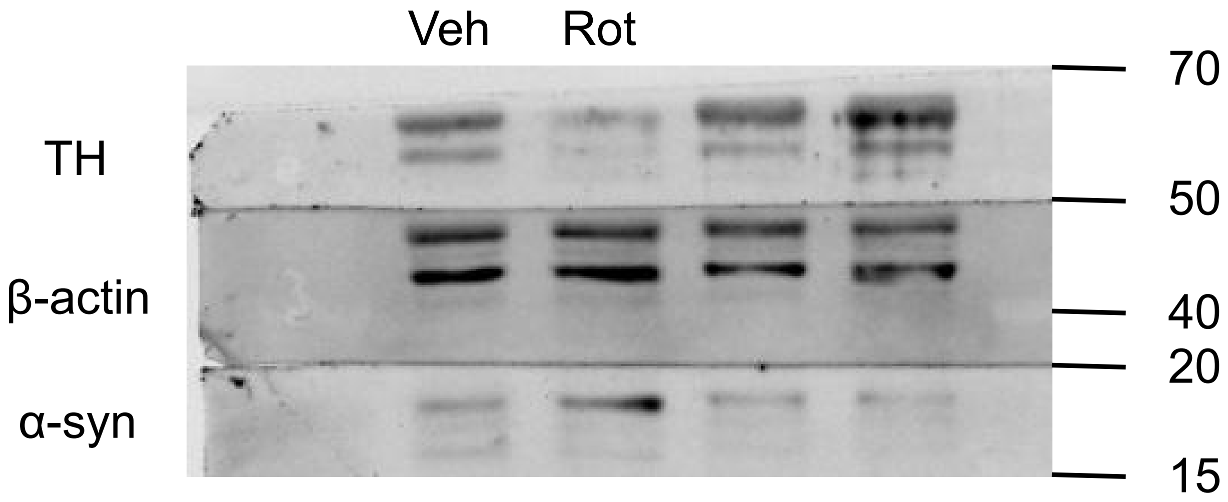

Original image of Western Blot in Fig1. C and Fig3. A

Fig2. F

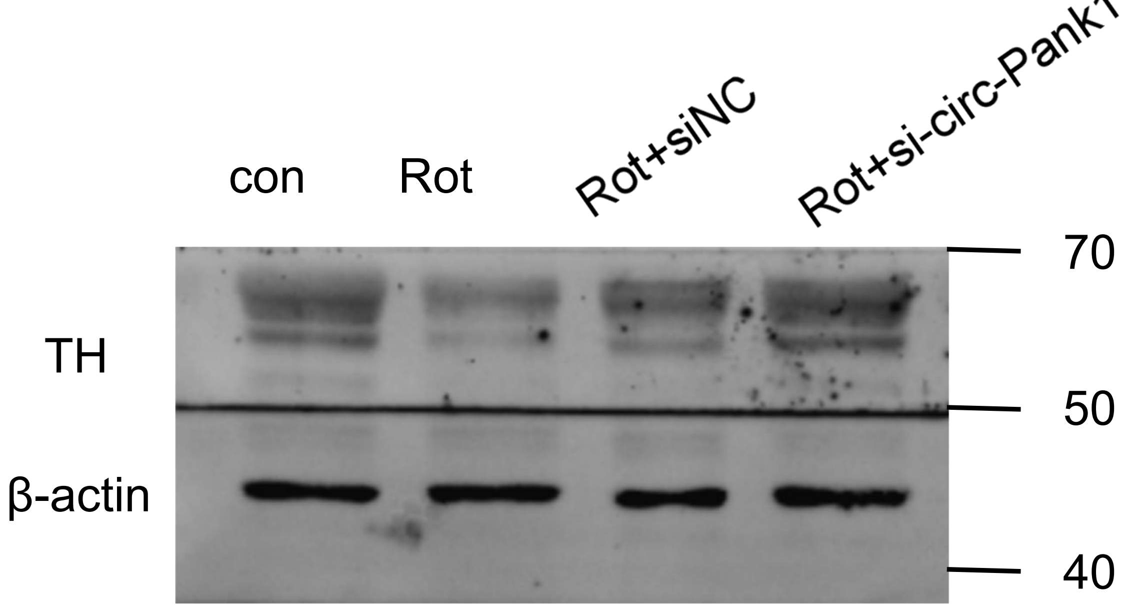

Original image of Western Blot in Fig2. F

Fig3. B

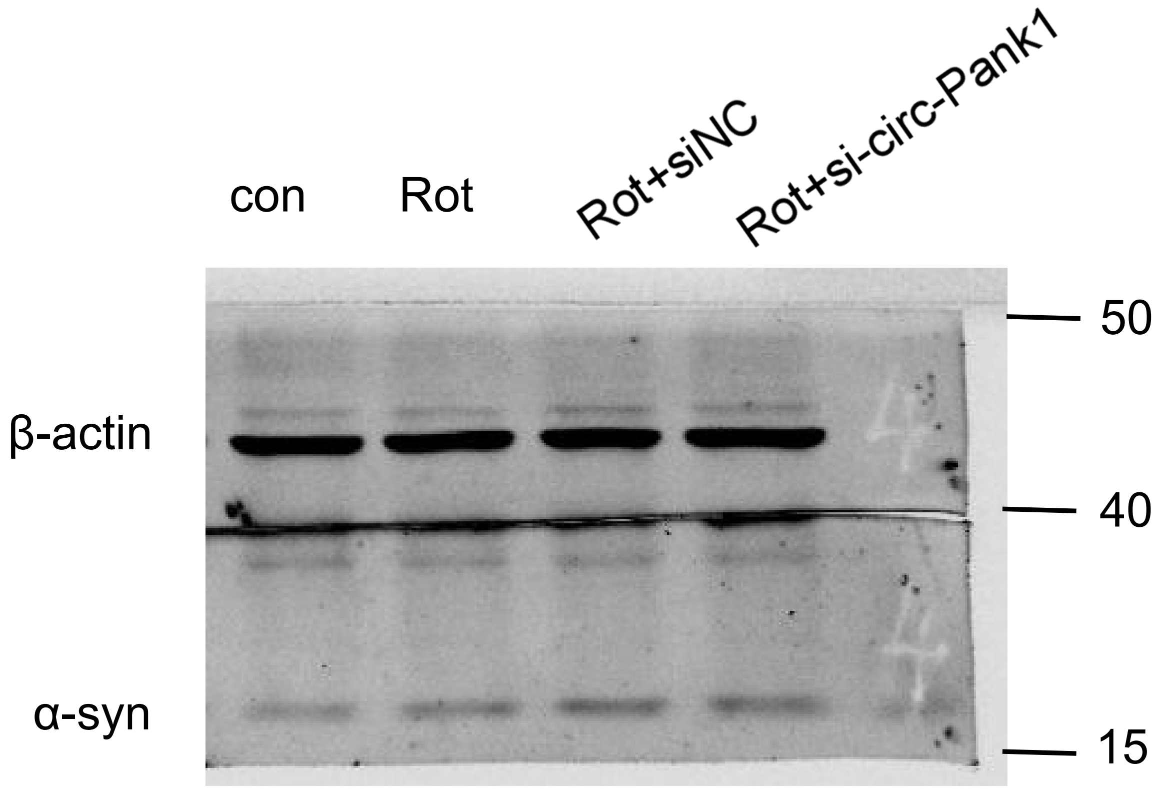

Original image of Western Blot in Fig3. B

Fig4. F

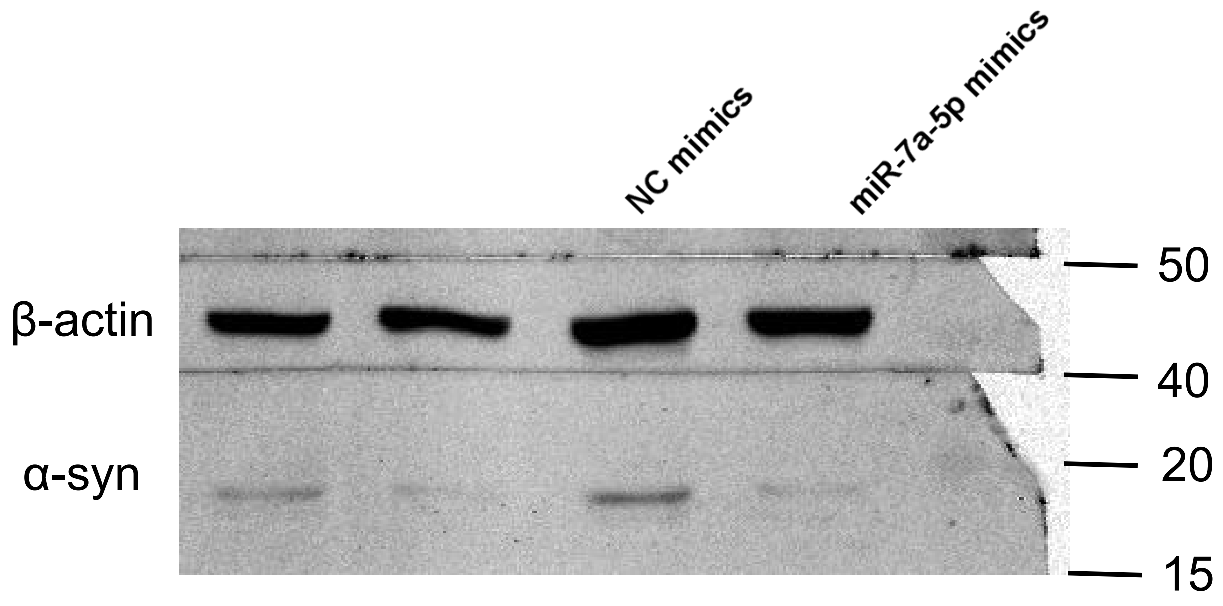

Original image of Western Blot in Fig4. F

Fig5. A

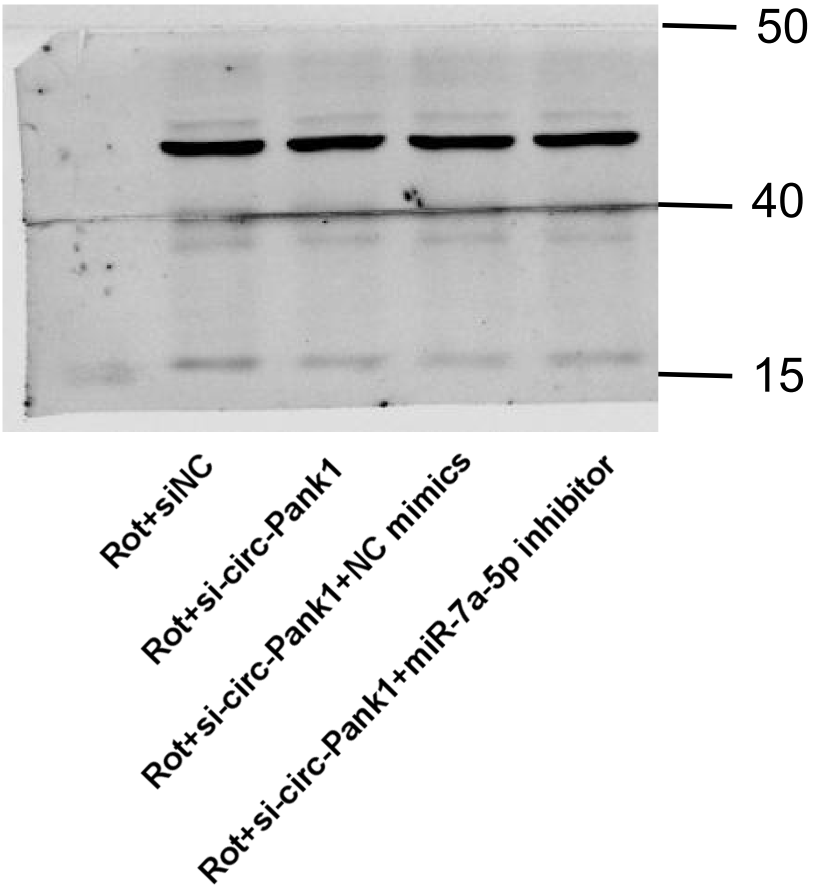

Fig5. B

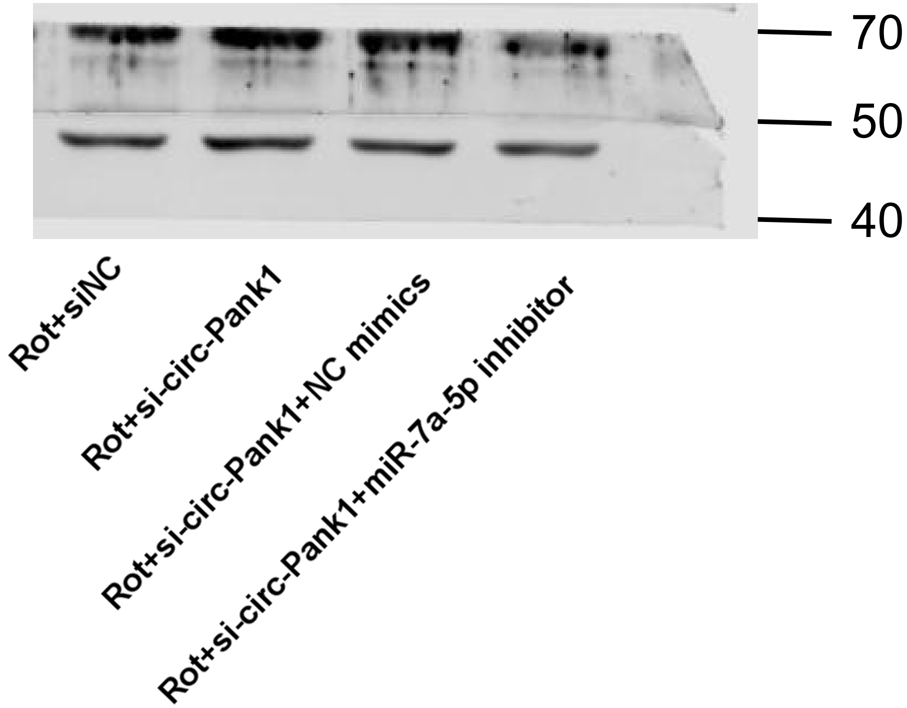

Original image of Western Blot in Fig5. A and Fig5. B

Fig6. G Fig6. I

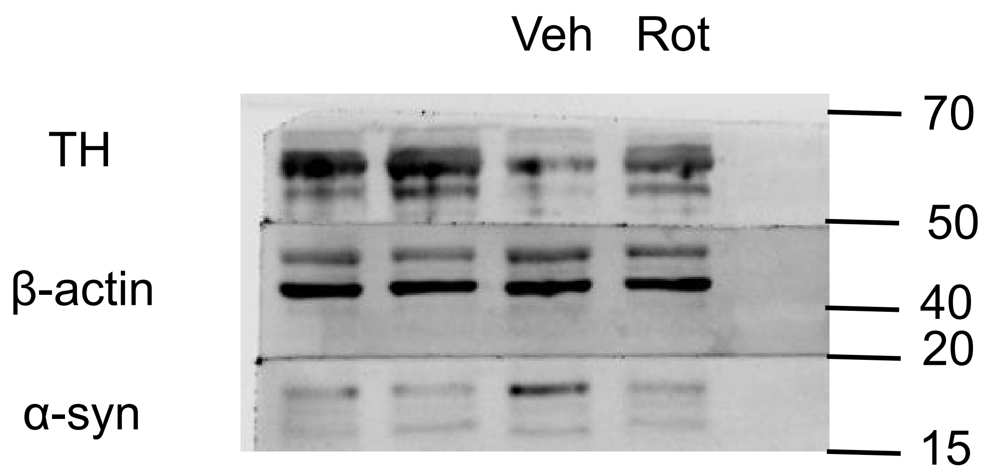

Original image of Western Blot in Fig6. G and Fig6. I
